# Supplementary material for: Big trees drive forest structure patterns across a lowland Amazon regrowth gradient
Source: Sci Rep. 2021 Feb 9;11:3380. doi: 10.1038/s41598-021-83030-5 (PMC7873124; doi:10.1038/s41598-021-83030-5)
Supplement: Supplementary file 1 — Supplementary Information 1 [file 41598_2021_83030_MOESM1_ESM.docx]

Big trees drive forest structure patterns across a lowland Amazon regrowth gradient

Tassiana Maylla Fontoura Caron^1^, Victor Juan Ulises Rodriguez Chuma^1,2^, Alexander Arévalo-Sandi^3,4^, Darren Norris^1, 3, 5^ *

^1^ Programa de Pós-Graduação em Biodiversidade Tropical, Universidade Federal do Amapá (UNIFAP), Rod. Juscelino Kubitscheck, Km 02, 68902-280, Macapá, AP, Brazil.

^2^ Facultad de Ciencias Forestales, Universidad Nacional de la Amazonia Peruana (UNAP), Pevas 5ta cuadra, Iquitos, Perú.

^3^ Programa de Pós-graduação em Ecologia, Instituto Nacional de Pesquisas da Amazônia (INPA), Av. André Araújo 2936, Petrópolis 69067-375 Manaus, AM, Brazil.

^4^ Amazonian Mammals Research Group. Av. André Araújo 2936, Petrópolis 69067-375 Manaus, AM, Brazil.

^5^ Coordenação de Ciências Ambientais, Universidade Federal do Amapá (UNIFAP), Rod. Juscelino Kubitschek Km 02, 68902-280 Macapá, AP, Brazil

*^*^ Corresponding author at: Coordenação de Ciências Ambientais, Universidade Federal do Amapá (UNIFAP), Macapá, Brazil. Email address: dnorris75@gmail.com (D. Norris).

Supplementary Table S1

Table S1: Generalized Linear Model values.

|  | **All trees** | | | | | | **Large trees** | | | | | |
| --- | --- | --- | --- | --- | --- | --- | --- | --- | --- | --- | --- | --- |
|  | **Full** | | | **Best** | | | **Full** | | | **Best** | | |
| *Predictors* | *Est.* | *CI* | *p* | *Est.* | *CI* | *p* | *Est.* | *CI* | *p* | *Est.* | *CI* | *p* |
| (Intercept) | 0.46 | 0.40–0.53 | **<0.001** | 0.46 | 0.43–0.50 | **<0.001** | 0.66 | 0.50–0.81 | **<0.001** | 0.64 | 0.55–0.76 | **<0.001** |
| Last use | 0.00 | -0.00–0.00 | 0.933 |  |  |  | -0.00 | -0.01–0.01 | 0.896 |  |  |  |
| Regrowth (late v early) | -0.01 | -0.10–0.07 | 0.826 | -0.03 | -0.08–0.01 | 0.176 | -0.04 | -0.24–0.17 | 0.710 | -0.05 | -0.20–0.08 | 0.441 |
| Regrowth (late v pasture) | -0.00 | -0.09–0.08 | 0.966 | -0.03 | -0.07–0.02 | 0.245 | -0.08 | -0.27–0.10 | 0.355 | -0.07 | -0.21–0.06 | 0.311 |
| Plot (control v regrowth) | 0.14 | 0.02–0.28 | **0.050** | 0.08 | 0.02–0.14 | **0.022** | 0.02 | -0.40–0.44 | 0.917 | 0.53 | 0.24–1.14 | **0.016** |
| Interaction (Last use * early) | -0.01 | -0.02–0.01 | 0.467 |  |  |  | -0.01 | -0.05–0.03 | 0.525 |  |  |  |
| Interaction (Last use * pasture) | -0.00 | -0.01–0.00 | 0.386 |  |  |  | -0.00 | -0.01–0.01 | 0.986 |  |  |  |
| Interaction (Last use * Plot) | -0.00 | -0.01–0.00 | 0.280 |  |  |  | 0.03 | -0.04–0.10 | 0.339 |  |  |  |
| Interaction (Plot * early) | 0.03 | -0.11–0.17 | 0.624 | 0.09 | -0.01–0.19 | 0.090 | 46.73 |  | 0.999 |  |  |  |
| Interaction (Plot * pasture) | 0.15 | 0.02–0.29 | **0.040** | 0.17 | 0.06–0.30 | **0.011** | 42.03 |  | 0.999 |  |  |  |
| Observations | 30 | | | 30 | | | 30 | | | 30 | | |
| R^2^Nagelkerke | 0.83 | | | 0.80 | | | 0.81 | | | 0.74 | | |
| AIC | 229.40 | | | 225.62 | | | 140.45 | | | 132.02 | | |

Supplementary Table S2

Generalized Linear Models fitted to explain forest structure in 30 plots (15 control and 15 regrowth). Model summaries for responses of five structural attributes: (a) The number of all trees ≥ 10 cm DBH ; (b) Proportion of small (DBH 10 – 20 cm) trees; (c) Proportion of large (DBH >60 cm) trees; (d) Basal area of all trees; (e) Basal area of large trees. Showing slope estimates (“Est”) for 10 variables in 5 models (time, regrowth class, mammal diversity, hydrography and topography). “NE” denotes cases when values could not be reliably estimated. The time model included years since the regrowth site was opened and years since last use. The regrowth model included sites grouped into three regrowth classes (pasture, early-regrowth and late-regrowth). Topography included altitude (masl) and slope. Hydrography was modelled with TWI (Topographic wetness index), DND (Distance to Network Drainage) calculated from the interaction between HAND (Height above network drainage) and HDND (Horizontal distance to network drainage). Mammal diversity was obtained from camera-traps and quantified as species richness and Functional Dispersion (FDis).

1. The number of all trees DBH ≥ 10 cm

|  | **Time** | | | **Regrowth class** | | | **Mammals** | | | **Hydrography** | | | **Topography** | | |
| --- | --- | --- | --- | --- | --- | --- | --- | --- | --- | --- | --- | --- | --- | --- | --- |
| *Predictors* | *Est* | *CI* | *p* | *Est* | *CI* | *p* | *Est* | *CI* | *p* | *Est* | *CI* | *p* | *Est* | *CI* | *p* |
| (Intercept) | 35.4 | 15.9–55.0 | **0.002** | 35.2 | 27.3–43.1 | **<0.001** | 25.04 | 5.31–44.77 | **0.021** | 20.04 | -25.26–65.35 | 0.395 | 33.66 | -67.46–134.78 | 0.521 |
| Plot type | -5.2 | -32.9–22.4 | 0.714 | 6.6 | -4.6–17.8 | 0.257 | -8.48 | -29.64–12.67 | 0.440 | -95.06 | -201.10–10.97 | 0.093 | -402.66 | -1045.25–239.93 | 0.232 |
| Since first open | -0.2 | -1.2–0.9 | 0.764 |  |  |  |  |  |  |  |  |  |  |  |  |
| Since last use | 0.1 | -2.7–2.8 | 0.954 |  |  |  |  |  |  |  |  |  |  |  |  |
| Plot *First | -0.4 | -1.9–1.1 | 0.573 |  |  |  |  |  |  |  |  |  |  |  |  |
| Plot *Last | -0.8 | -4.7–3.1 | 0.703 |  |  |  |  |  |  |  |  |  |  |  |  |
| First *Last | 0.0 | -0.1–0.1 | 0.963 |  |  |  |  |  |  |  |  |  |  |  |  |
| Plot *First *Last | 0.1 | -0.1–0.3 | 0.388 |  |  |  |  |  |  |  |  |  |  |  |  |
| Regrowth (Late v Early) |  |  |  | -1.2 | -12.4–10.0 | 0.835 |  |  |  |  |  |  |  |  |  |
| Regrowth (Late v Pasture) |  |  |  | -2.4 | -13.6–8.8 | 0.677 |  |  |  |  |  |  |  |  |  |
| Plot *Early |  |  |  | -14.0 | -29.8–1.8 | 0.095 |  |  |  |  |  |  |  |  |  |
| Plot*Pasture |  |  |  | -23.2 | -39.0–-7.4 | **0.008** |  |  |  |  |  |  |  |  |  |
| Species.richness |  |  |  |  |  |  | 2.9 | -7.0–12.8 | 0.571 |  |  |  |  |  |  |
| Functional.dispersion |  |  |  |  |  |  | 41.7 | -78.5–162.0 | 0.503 |  |  |  |  |  |  |
| Plot *Species.richness |  |  |  |  |  |  | 15.1 | 1.6–28.5 | **0.039** |  |  |  |  |  |  |
| Plot *FDis |  |  |  |  |  |  | -37.0 | -174.8–100.75 | 0.604 |  |  |  |  |  |  |
| Richness *FDis |  |  |  |  |  |  | -11.6 | -52.86–29.65 | 0.587 |  |  |  |  |  |  |
| Plot *Richness *FDis |  |  |  |  |  |  | -36.2 | -88.80–16.5 | 0.192 |  |  |  |  |  |  |
| TWI |  |  |  |  |  |  |  |  |  | 1.8 | -4.2–7.8 | 0.561 |  |  |  |
| DND |  |  |  |  |  |  |  |  |  | -6.6 | -42.1–29.0 | 0.722 |  |  |  |
| Plot * TWI |  |  |  |  |  |  |  |  |  | 12.7 | -2.0–27.3 | 0.105 |  |  |  |
| Plot * DND |  |  |  |  |  |  |  |  |  | -155.8 | -285.0–-25.3 | **0.029** |  |  |  |
| TWI * DND |  |  |  |  |  |  |  |  |  | 1.6 | -4.6–7.7 | 0.623 |  |  |  |
| Plot * TWI* DND |  |  |  |  |  |  |  |  |  | 20.4 | 0.9–39.9 | 0.053 |  |  |  |
| Altitude |  |  |  |  |  |  |  |  |  |  |  |  | -0.0 | -1.0–1.0 | 0.980 |
| Slope |  |  |  |  |  |  |  |  |  |  |  |  | -1.7 | -16.9–13.8 | 0.845 |
| Plot *Altitude |  |  |  |  |  |  |  |  |  |  |  |  | 3.7 | -2.4–9.7 | 0.250 |
| Plot * Slope |  |  |  |  |  |  |  |  |  |  |  |  | 37.9 | -70.3–146.2 | 0.499 |
| Altitude * Slope |  |  |  |  |  |  |  |  |  |  |  |  | 0.0 | -0.1–0.2 | 0.813 |
| Plot *Altitude* Slope |  |  |  |  |  |  |  |  |  |  |  |  | -0.34 | -1.4–0.7 | 0.522 |
| Observations | 30 | | | 30 | | | 30 | | | 30 | | | 30 | | |
| R^2^ Nagelkerke | 1.000 | | | 1.000 | | | 1.000 | | | 1.000 | | | 1.000 | | |
| Deviance | 2206.473 | | | 1942.400 | | | 1883.533 | | | 2095.536 | | | 1438.744 | | |
| AIC | 232.075 | | | 224.251 | | | 227.328 | | | 230.527 | | | 219.246 | | |
| log-Likelihood | -107.037 | | | -105.125 | | | -104.664 | | | -106.264 | | | -100.623 | | |

1. Proportion of small (DBH 10 – 20 cm) trees

|  | **Time** | | | **Regrowth class** | | | **Mammals** | | | **Hydrography** | | | **Topography** | | |
| --- | --- | --- | --- | --- | --- | --- | --- | --- | --- | --- | --- | --- | --- | --- | --- |
| *Predictors* | *OddsRatios* | *CI* | *p* | *OddsRatios* | *CI* | *p* | *OddsRatios* | *CI* | *p* | *OddsRatios* | *CI* | *p* | *OddsRatios* | *CI* | *p* |
| (Intercept) | 1.1 | 0.6–2.2 | 0.704 | 1.6 | 1.2–2.2 | **0.002** | 1.1 | 0.5–2.5 | 0.778 | 0.5 | 0.11–2.7 | 0.461 | 5.2 | 0.1–515.3 | 0.475 |
| Plot type | 38.4 | 8.6–236.0 | **<0.001** | 3.7 | 2.0–5.1 | **<0.001** | 5.7 | 2.1–15.3 | **0.001** | 0.4 | 0.00–66.8 | 0.713 | 126.0 | 0.0–313.0 | 0.713 |
| Since first open | 1.0 | 1.0–1.1 | 0.336 |  |  |  |  |  |  |  |  |  |  |  |  |
| Since last use | 1.0 | 0.9–1.1 | 0.834 |  |  |  |  |  |  |  |  |  |  |  |  |
| Plot *First | 0.9 | 0.8–1.0 | **0.015** |  |  |  |  |  |  |  |  |  |  |  |  |
| Plot *Last | 0.8 | 0.7–1.0 | **0.017** |  |  |  |  |  |  |  |  |  |  |  |  |
| First *Last | 1.0 | 1.0–1.0 | 0.841 |  |  |  |  |  |  |  |  |  |  |  |  |
| Plot *First *Last | 1.0 | 1.0–1.0 | **0.029** |  |  |  |  |  |  |  |  |  |  |  |  |
| Regrowth (Late v Early) |  |  |  | 0.8 | 0.5–1.2 | 0.302 |  |  |  |  |  |  |  |  |  |
| Regrowth (Late v Pasture) |  |  |  | 1.0 | 0.6–1.5 | 0.856 |  |  |  |  |  |  |  |  |  |
| Plot *Early |  |  |  | 1.7 | 0.8–3.6 | 0.190 |  |  |  |  |  |  |  |  |  |
| Plot*Pasture |  |  |  | 1.0 | 0.4–2.2 | 0.938 |  |  |  |  |  |  |  |  |  |
| Species.richness |  |  |  |  |  |  | 1.2 | 0.8–1.8 | 0.284 |  |  |  |  |  |  |
| Functional.dispersion |  |  |  |  |  |  | 2.3 | 0.0–256.3 | 0.724 |  |  |  |  |  |  |
| Plot *Species.richness |  |  |  |  |  |  | 1.3 | 0.7–2.5 | 0.415 |  |  |  |  |  |  |
| Plot *FDis |  |  |  |  |  |  | 0.0 | 0.0–0.3 | **0.020** |  |  |  |  |  |  |
| Richness *FDis |  |  |  |  |  |  | 0.5 | 0.1–2.4 | 0.389 |  |  |  |  |  |  |
| Plot *Richness *FDis |  |  |  |  |  |  | 1.7 | 0.1–22.2 | 0.702 |  |  |  |  |  |  |
| TWI |  |  |  |  |  |  |  |  |  | 1.1 | 1.0–1.4 | 0.210 |  |  |  |
| DND |  |  |  |  |  |  |  |  |  | 0.4 | 0.1–1.5 | 0.200 |  |  |  |
| Plot * TWI |  |  |  |  |  |  |  |  |  | 1.3 | 0.6–2.6 | 0.464 |  |  |  |
| Plot * DND |  |  |  |  |  |  |  |  |  | 0.0 | 0.0–12.4 | 0.211 |  |  |  |
| TWI * DND |  |  |  |  |  |  |  |  |  | 1.2 | 0.9–1.5 | 0.169 |  |  |  |
| Plot * TWI* DND |  |  |  |  |  |  |  |  |  | 1.5 | 0.6–3.8 | 0.411 |  |  |  |
| Altitude |  |  |  |  |  |  |  |  |  |  |  |  | 1.0 | 1.0–1.0 | 0.775 |
| Slope |  |  |  |  |  |  |  |  |  |  |  |  | 0.6 | 0.3–1.2 | 0.153 |
| Plot *Altitude |  |  |  |  |  |  |  |  |  |  |  |  | 0.9 | 0.6–1.5 | 0.803 |
| Plot * Slope |  |  |  |  |  |  |  |  |  |  |  |  | 0.7 | 0.0–160.2 | 0.920 |
| Altitude * Slope |  |  |  |  |  |  |  |  |  |  |  |  | 1.0 | 1.0–1.0 | 0.190 |
| Plot *Altitude* Slope |  |  |  |  |  |  |  |  |  |  |  |  | 1.0 | 0.9–1.1 | 0.965 |
| Observations | 30 | | | 30 | | | 30 | | | 30 | | | 30 | | |
| R^2^Tjur | 0.133 | | | 0.111 | | | 0.113 | | | 0.188 | | | 0.192 | | |
| Deviance | 46.970 | | | 60.088 | | | 45.259 | | | 39.873 | | | 33.410 | | |
| AIC | 158.064 | | | 167.181 | | | 156.352 | | | 150.967 | | | 144.503 | | |
| log-Likelihood | -71.032 | | | -77.591 | | | -70.176 | | | -67.483 | | | -64.252 | | |

1. Proportion of large (DBH >60 cm) trees

|  | **Time** | | | **Regrowth class** | | | **Mammals** | | | **Hydrography** | | | **Topography** | | |
| --- | --- | --- | --- | --- | --- | --- | --- | --- | --- | --- | --- | --- | --- | --- | --- |
| *Predictors* | *Odds Ratios* | *CI* | *p* | *Odds Ratios* | *CI* | *p* | *Odds Ratios* | *CI* | *p* | *Odds Ratios* | *CI* | *p* | *Odds Ratios* | *CI* | *p* |
| (Intercept) | 0.0 | 0.0–0.1 | **<0.001** | 0.0 | 0.0–0.1 | **<0.001** | 0.1 | 0.0–0.5 | **0.004** | 0.0 | 0.0–0.8 | **0.032** | 6.43 | NE | 0.647 |
| Plot type | 0.4 | 0.0–54.2 | 0.714 | 0.2 | 0.0–0.9 | **0.042** | 0.0 | 0.0–0.9 | **0.040** | 3.9 | NE | 0.887 | NE | NE | 0.830 |
| Since first open | 1.1 | 1.0–1.2 | 0.071 |  |  |  |  |  |  |  |  |  |  |  |  |
| Since last use | 1.2 | 1.0–1.5 | 0.088 |  |  |  |  |  |  |  |  |  |  |  |  |
| Plot *First | 1.0 | 0.7–1.2 | 0.685 |  |  |  |  |  |  |  |  |  |  |  |  |
| Plot *Last | 0.8 | 0.4–1.6 | 0.492 |  |  |  |  |  |  |  |  |  |  |  |  |
| First *Last | 1.0 | 1.0–1.0 | 0.060 |  |  |  |  |  |  |  |  |  |  |  |  |
| Plot *First *Last | 1.0 | 1.0–1.0 | 0.505 |  |  |  |  |  |  |  |  |  |  |  |  |
| Regrowth (Late v Early) |  |  |  | 1.5 | 0.6–3.8 | 0.417 |  |  |  |  |  |  |  |  |  |
| Regrowth (Late v Pasture) |  |  |  | 2.4 | 1.0–5.8 | 0.060 |  |  |  |  |  |  |  |  |  |
| Plot *Early |  |  |  | 0.3 | 0.0–9.6 | 0.526 |  |  |  |  |  |  |  |  |  |
| Plot*Pasture |  |  |  | 0.3 | 0.0–9.7 | 0.530 |  |  |  |  |  |  |  |  |  |
| Species.richness |  |  |  |  |  |  | 0.9 | 0.4–1.8 | 0.653 |  |  |  |  |  |  |
| Functional.dispersion |  |  |  |  |  |  | 0.6 | NE | 0.921 |  |  |  |  |  |  |
| Plot *Species.richness |  |  |  |  |  |  | 0.1 | 0.0–5.3 | 0.271 |  |  |  |  |  |  |
| Plot *FDis |  |  |  |  |  |  | 165.7 | NE | 0.540 |  |  |  |  |  |  |
| Richness *FDis |  |  |  |  |  |  | 0.9 | 0.0–17.0 | 0.936 |  |  |  |  |  |  |
| Plot *Richness *FDis |  |  |  |  |  |  | 1114.3 | NE | 0.253 |  |  |  |  |  |  |
| TWI |  |  |  |  |  |  |  |  |  | 1.1 | 0.7–1.6 | 0.659 |  |  |  |
| DND |  |  |  |  |  |  |  |  |  | 0.6 | 0.1–4.9 | 0.593 |  |  |  |
| Plot * TWI |  |  |  |  |  |  |  |  |  | 0.6 | 0.0–7.5 | 0.669 |  |  |  |
| Plot * DND |  |  |  |  |  |  |  |  |  | NE | NE | 0.393 |  |  |  |
| TWI * DND |  |  |  |  |  |  |  |  |  | 1.1 | 0.8–1.6 | 0.562 |  |  |  |
| Plot * TWI* DND |  |  |  |  |  |  |  |  |  | 0.3 | 0.0–10.4 | 0.462 |  |  |  |
| Altitude |  |  |  |  |  |  |  |  |  |  |  |  | 0.96 | 0.89–1.03 | 0.265 |
| Slope |  |  |  |  |  |  |  |  |  |  |  |  | 0.63 | 0.18–2.16 | 0.464 |
| Plot *Altitude |  |  |  |  |  |  |  |  |  |  |  |  | 0.81 | 0.13–5.00 | 0.821 |
| Plot * Slope |  |  |  |  |  |  |  |  |  |  |  |  | 0.12 | NE | 0.897 |
| Altitude * Slope |  |  |  |  |  |  |  |  |  |  |  |  | 1.00 | 0.99–1.01 | 0.406 |
| Plot *Altitude* Slope |  |  |  |  |  |  |  |  |  |  |  |  | 1.02 | 0.75–1.38 | 0.906 |
| Observations | 30 | | | 30 | | | 30 | | | 30 | | | 30 | | |
| R^2^ Tjur | 0.027 | | | 0.008 | | | 0.001 | | | 0.028 | | | 0.026 | | |
| Deviance | 22.439 | | | 23.760 | | | 22.382 | | | 28.416 | | | 27.326 | | |
| AIC | 72.554 | | | 69.778 | | | 72.532 | | | 78.313 | | | 77.272 | | |
| log-Likelihood | -28.277 | | | -28.889 | | | -28.266 | | | -31.156 | | | -30.636 | | |

1. Basal area of all trees

|  | **Time** | | | **Regrowth class** | | | **Mammals** | | | **Hydrography** | | | **Topography** | | |
| --- | --- | --- | --- | --- | --- | --- | --- | --- | --- | --- | --- | --- | --- | --- | --- |
| *Predictors* | *Est* | *CI* | *p* | *Est* | *CI* | *p* | *Est* | *CI* | *p* | *Est* | *CI* | *p* | *Est* | *CI* | *p* |
| (Intercept) | 0.45 | 0.39–0.52 | **<0.001** | 0.46 | 0.43–0.50 | **<0.001** | 0.43 | 0.36–0.51 | **<0.001** | 0.48 | 0.31–0.65 | **<0.001** | 0.48 | 0.10–0.90 | **0.028** |
| Plot type | 0.21 | 0.03–0.43 | **0.046** | 0.08 | 0.02–0.14 | **0.022** | 0.25 | 0.13–0.38 | **0.001** | 0.35 | -0.59–1.47 | 0.500 | 4.85 | -0.94–10.40 | 0.106 |
| Since first open | -0.00 | -0.00–0.00 | 0.956 |  |  |  |  |  |  |  |  |  |  |  |  |
| Since last use | -0.00 | -0.01–0.00 | 0.341 |  |  |  |  |  |  |  |  |  |  |  |  |
| Plot *First | -0.00 | -0.01–0.01 | 0.790 |  |  |  |  |  |  |  |  |  |  |  |  |
| Plot *Last | 0.00 | -0.02–0.02 | 0.998 |  |  |  |  |  |  |  |  |  |  |  |  |
| First *Last | 0.00 | -0.00–0.00 | 0.331 |  |  |  |  |  |  |  |  |  |  |  |  |
| Plot *First *Last | -0.00 | -0.00–0.00 | 0.719 |  |  |  |  |  |  |  |  |  |  |  |  |
| Regrowth (Late v Early) |  |  |  | -0.03 | -0.08–0.01 | 0.176 |  |  |  |  |  |  |  |  |  |
| Regrowth (Late v Pasture) |  |  |  | -0.03 | -0.07–0.02 | 0.245 |  |  |  |  |  |  |  |  |  |
| Plot *Early |  |  |  | 0.09 | -0.01–0.19 | 0.090 |  |  |  |  |  |  |  |  |  |
| Plot*Pasture |  |  |  | 0.17 | 0.06–0.30 | **0.011** |  |  |  |  |  |  |  |  |  |
| Species.richness |  |  |  |  |  |  | 0.01 | -0.03–0.05 | 0.624 |  |  |  |  |  |  |
| Functional.dispersion |  |  |  |  |  |  | -0.04 | -0.53–0.42 | 0.855 |  |  |  |  |  |  |
| Plot *Species.richness |  |  |  |  |  |  | -0.06 | -0.15–0.03 | 0.192 |  |  |  |  |  |  |
| Plot *FDis |  |  |  |  |  |  | -0.53 | -1.28–0.22 | 0.178 |  |  |  |  |  |  |
| Richness *FDis |  |  |  |  |  |  | -0.01 | -0.17–0.15 | 0.900 |  |  |  |  |  |  |
| Plot *Richness *FDis |  |  |  |  |  |  | 0.22 | -0.11–0.57 | 0.210 |  |  |  |  |  |  |
| TWI |  |  |  |  |  |  |  |  |  | -0.01 | -0.03–0.02 | 0.634 |  |  |  |
| DND |  |  |  |  |  |  |  |  |  | 0.05 | -0.09–0.17 | 0.499 |  |  |  |
| Plot * TWI |  |  |  |  |  |  |  |  |  | -0.03 | -0.18–0.10 | 0.679 |  |  |  |
| Plot * DND |  |  |  |  |  |  |  |  |  | 0.46 | -0.74–1.86 | 0.486 |  |  |  |
| TWI * DND |  |  |  |  |  |  |  |  |  | -0.01 | -0.03–0.02 | 0.468 |  |  |  |
| Plot * TWI* DND |  |  |  |  |  |  |  |  |  | -0.06 | -0.26–0.12 | 0.553 |  |  |  |
| Altitude |  |  |  |  |  |  |  |  |  |  |  |  | 0.00 | -0.00–0.00 | 0.994 |
| Slope |  |  |  |  |  |  |  |  |  |  |  |  | -0.02 | -0.08–0.03 | 0.412 |
| Plot *Altitude |  |  |  |  |  |  |  |  |  |  |  |  | -0.04 | -0.10–0.01 | 0.126 |
| Plot * Slope |  |  |  |  |  |  |  |  |  |  |  |  | -0.48 | -1.33–0.43 | 0.297 |
| Altitude * Slope |  |  |  |  |  |  |  |  |  |  |  |  | 0.00 | -0.00–0.00 | 0.473 |
| Plot *Altitude* Slope |  |  |  |  |  |  |  |  |  |  |  |  | 0.00 | -0.00–0.01 | 0.316 |
| Observations | 30 | | | 30 | | | 30 | | | 30 | | | 30 | | |
| R^2^ Nagelkerke | 0.997 | | | 0.998 | | | 0.997 | | | 0.996 | | | 0.999 | | |
| Deviance | 74.501 | | | 65.927 | | | 68.631 | | | 82.585 | | | 47.959 | | |
| AIC | 233.200 | | | 225.615 | | | 230.793 | | | 236.227 | | | 220.376 | | |

1. Basal area of large trees

|  | **Time** | | | **Regrowth class** | | | **Mammals** | | | **Hydrography** | | | **Topography** | | |
| --- | --- | --- | --- | --- | --- | --- | --- | --- | --- | --- | --- | --- | --- | --- | --- |
| *Predictors* | *Est* | *CI* | *p* | *Est* | *CI* | *p* | *Est* | *CI* | *p* | *Est* | *CI* | *p* | *Est* | *CI* | *p* |
| (Intercept) | 0.7 | 0.5–0.9 | **<0.001** | 0.7 | 0.6–0.7 | **<0.001** | 0.7 | 0.4–0.7 | **<0.001** | 0.7 | 0.4–1.0 | **<0.001** | 0.5 | -0.53–1.71 | 0.406 |
| Plot type | 535.7 | NE | 0.998 | 0.3 | -0.0–0.6 | 0.051 | 62.9 | NE | 0.999 | NE | NE | 0.823 | -31.5 | -204.4–39.4 | 0.552 |
| Since first open | -0.0 | -0.0–0.0 | 0.303 |  |  |  |  |  |  |  |  |  |  |  |  |
| Since last use | -0.0 | -0.0–0.0 | 0.186 |  |  |  |  |  |  |  |  |  |  |  |  |
| Plot *First | -26.7 | NE | 0.998 |  |  |  |  |  |  |  |  |  |  |  |  |
| Plot *Last | -27.4 | NE | 0.998 |  |  |  |  |  |  |  |  |  |  |  |  |
| First *Last | 0.0 | -0.0–0.0 | 0.159 |  |  |  |  |  |  |  |  |  |  |  |  |
| Plot *First *Last | 1.4 | -1447.1–1449.9 | 0.998 |  |  |  |  |  |  |  |  |  |  |  |  |
| Regrowth (Late v Early) |  |  |  | -0.1 | -0.2–0.0 | 0.189 |  |  |  |  |  |  |  |  |  |
| Regrowth (Late v Pasture) |  |  |  | -0.1 | -0.2–0.0 | 0.103 |  |  |  |  |  |  |  |  |  |
| Plot *Early |  |  |  | 39.5 | NE | 0.998 |  |  |  |  |  |  |  |  |  |
| Plot*Pasture |  |  |  | 39.5 | NE | 0.998 |  |  |  |  |  |  |  |  |  |
| Species.richness |  |  |  |  |  |  | 0.0 | -0.1–0.1 | 0.762 |  |  |  |  |  |  |
| Functional.dispersion |  |  |  |  |  |  | -0.1 | -1.0–0.8 | 0.811 |  |  |  |  |  |  |
| Plot *Species.richness |  |  |  |  |  |  | 89.8 | NE | 0.999 |  |  |  |  |  |  |
| Plot *FDis |  |  |  |  |  |  | -212.3 | NE | 0.999 |  |  |  |  |  |  |
| Richness *FDis |  |  |  |  |  |  | 0.0 | -0.3–0.3 | 0.859 |  |  |  |  |  |  |
| Plot *Richness *FDis |  |  |  |  |  |  | -252.1 | NE | 0.999 |  |  |  |  |  |  |
| TWI |  |  |  |  |  |  |  |  |  | -0.02 | -0.1–0.0 | 0.392 |  |  |  |
| DND |  |  |  |  |  |  |  |  |  | 0.11 | -0.1–0.3 | 0.273 |  |  |  |
| Plot * TWI |  |  |  |  |  |  |  |  |  | -63173.6 | NE | 0.823 |  |  |  |
| Plot * DND |  |  |  |  |  |  |  |  |  | 976655.4 | NE | 0.823 |  |  |  |
| TWI * DND |  |  |  |  |  |  |  |  |  | -0.02 | -0.1–0.0 | 0.239 |  |  |  |
| Plot * TWI* DND |  |  |  |  |  |  |  |  |  | -96752.4 | NE | 0.823 |  |  |  |
| Altitude |  |  |  |  |  |  |  |  |  |  |  |  | 0.0 | -0.0–0.0 | 0.765 |
| Slope |  |  |  |  |  |  |  |  |  |  |  |  | -0.0 | -0.2–0.1 | 0.861 |
| Plot *Altitude |  |  |  |  |  |  |  |  |  |  |  |  | 0.3 | -0.4–1.9 | 0.550 |
| Plot * Slope |  |  |  |  |  |  |  |  |  |  |  |  | 5.7 | -6.2–36.0 | 0.536 |
| Altitude * Slope |  |  |  |  |  |  |  |  |  |  |  |  | 0.0 | -0.0–0.0 | 0.913 |
| Plot *Altitude* Slope |  |  |  |  |  |  |  |  |  |  |  |  | -0.1 | -0.3–0.1 | 0.538 |
| Observations | 30 | | | 30 | | | 30 | | | 30 | | | 30 | | |
| R^2^ Nagelkerke | 1.000 | | | 1.000 | | | 1.000 | | | 1.000 | | | 1.000 | | |
| Deviance | 140.557 | | | 134.847 | | | 121.384 | | | 119.165 | | | 161.409 | | |
| AIC | 138.281 | | | 133.726 | | | 136.303 | | | 136.050 | | | 140.145 | | |
